# Unusual Epstein-Barr esophageal infection in an immunocompetent patient: a case report

**DOI:** 10.4076/1752-1947-3-7314

**Published:** 2009-06-29

**Authors:** Magdalini Pape, Kalliopi Mandraveli, Ioannis Sidiropoulos, Dimitrios Koliouskas, Stella Alexiou-Daniel, Filanthi Frantzidou

**Affiliations:** 1 Department of Microbiology, School of Medicine, Laboratory of Infectious Diseases, AHEPA University Hospital of Thessaloniki, Greece; 2 First Propedeutic Medical Department, AHEPA University Hospital, Aristotle University of Thessaloniki, Greece

## Abstract

**Introduction:**

Epstein-Barr virus esophagitis in an immunocompetent host is a rare entity. It represents either primary infection or reactivation and is usually characterized by acute onset and extensive ulcerative involvement of the upper and middle third of the esophagus.

**Case presentation:**

A case of Epstein-Barr virus esophagitis in a 27-year-old woman with no immunosuppressive factors, and having gastrointestinal symptoms is reported here. Using real-time polymerase chain reaction, biopsy and blood specimens were tested for candida and herpes viruses. Epstein-Barr virus DNA was detected in tissue samples. The patient was treated with acyclovir with resolution of the symptomatology.

**Conclusions:**

The prevalence of esophagitis remains undefined in both immunodeficient and immunocompetent individuals and should be taken into consideration in a patient presenting with esophageal symptoms. This case report stresses the role of Epstein-Barr virus infection in the pathogenesis of esophagitis, a rare condition in an immunocompetent host. In this setting, active infection may represent a primary infection or reactivation. Histopathological examination alone may miss the diagnosis, while polymerase chain reaction techniques optimize the diagnostic sensitivity, establish a diagnosis, and lead to an appropriate therapy.

## Introduction

Esophageal infections are a well known complication and a significant cause of morbidity in patients with an impaired immune system [1,2]. Patients at high risk are commonly HIV-infected and leukopenic individuals, recipients of transplants or recipients of immunosuppressive medications [3,4]. The most frequently identified esophageal pathogens are candida [1], cytomegalovirus (CMV) [5], and herpes simplex virus (HSV) [6]. Epstein-Barr virus (EBV) is an important human pathogen, causing a variety of syndromes. Infections of the gastrointestinal tract, a common form of disease of EBV, are usually manifest as colitis and Crohn's disease [7,8]. However, the prevalence of EBV esophagitis remains undefined in both immunodeficient and immunocompetent individuals and should be taken into consideration in a patient presenting with esophageal symptoms.

## Case presentation

A 27-year-old woman with no past medical history was admitted to the emergency department of our hospital with a 7-day history of dysphagia, especially for solid food, and odynophagia. She had no previous history of upper gastrointestinal complaints or any systemic symptoms. Her physical examination was normal as was an abdominal computed tomography (CT) scan. Routine admission laboratory studies were normal and serological tests (ELISA AXSYM Abbott) for anti-cytomegalovirus, anti-Epstein-Barr virus, anti-herpes simplex virus and anti-varicella zoster virus IgG/IgM antibodies excluded primary infection or reactivation. The serology results for EBV were: IgG (+) 125 IU/ml, IgM (−).

The patient underwent esophagoscopy, using an Olympus endoscope and multiple, well circumscribed ulcerations were identified in the upper and middle section of the esophagus. The ulcers were characterized as either shallow or of intermediate depth. A few deep ulcers were also seen. Viral infection was taken into consideration on the basis of the gastrointestinal symptoms and in the absence of any other demonstrable causes such as drug-induced esophagitis. Biopsy specimens were obtained from the identified ulcers and submitted for histopathologic evaluation. Tissue sections were also stained with hematoxylin and eosin. Microscopically, all specimens showed non-specific features of active esophagitis, including ulceration, neutrophilic and eosinophilic inflammation, and a basal cell hyperplasia. No viral inclusions, hyperchromaticity, or atypical mitoses were observed. Using real-time polymerase chain reaction (PCR) (artus LC PCR, QIAGEN), biopsy and blood specimens were tested for the most and less frequently identified esophageal pathogens (candida, CMV, HSV, EBV). No candida, CMV, HSV or EBV DNA was detected in blood samples. EBV DNA was detected only in tissue samples (1.6 × 10^3^ copies/ml). Therefore, the patient was started on treatment with acyclovir at an oral dosage of 800 mg, five times daily for 5 days, with good resolution of the symptomatology. Follow-up endoscopic examination showed a clear improvement. PCR on biopsy materials was once more performed, but no EBV genome could be detected. Blood samples were also obtained, but no serological evidence of infection was reported. The patient has subsequently remained symptom-free.

## Discussion

Herpes virus esophagitis is a well-known infectious complication in patients with impaired immune system, and has also been described as a self-limiting illness in immunocompetent patients. The herpes viruses so far related to esophagitis are CMV and HSV. Although a few reports have suggested the role of EBV in esophagitis, we believe that EBV infection may activate immune-mediated mechanisms eventually leading to tissue damage. This case report stresses the role of EBV infection in the pathogenesis of esophagitis, a rare condition in the immunocompetent host. In this setting, active infection may represent a primary infection or reactivation.

Histopathological examination alone may lead to incorrect diagnosis, while PCR techniques optimize the diagnostic sensitivity, establish a firm diagnosis and lead to an appropriate therapy.

## Conclusions

EBV infection should be considered in all symptomatic patients, immunocompetent or not, in whom esophageal ulceration is identified endoscopically and who are not being treated with immunosuppressants and/or corticosteroids. The PCR technique clearly establishes the diagnosis, leading to an appropriate treatment.

## Consent

Written informed consent was obtained from the patient for publication of this case report. A copy of the written consent is available for review by the Editor-in-Chief of this journal.

## Competing interests

The authors declare that they have no competing interests.

## Authors' contributions

PM is the primary contributing author. SI is a pathological specialist and was the medical internist responsible for the patient. All authors read and approved the final manuscript.
